# A multi-center preclinical study of gadoxetate DCE-MRI in rats as a biomarker of drug induced inhibition of liver transporter function

**DOI:** 10.1371/journal.pone.0197213

**Published:** 2018-05-17

**Authors:** Anastassia Karageorgis, Stephen C. Lenhard, Brittany Yerby, Mikael F. Forsgren, Serguei Liachenko, Edvin Johansson, Mark A. Pilling, Richard A. Peterson, Xi Yang, Dominic P. Williams, Sharon E. Ungersma, Ryan E. Morgan, Kim L. R. Brouwer, Beat M. Jucker, Paul D. Hockings

**Affiliations:** 1 Safety and ADME Translational Sciences, Drug Safety and Metabolism, AstraZeneca, Gothenburg, Sweden; 2 Bioimaging, Platform Technology and Sciences, GlaxoSmithKline, King of Prussia, Pennsylvania, United States of America; 3 Research Imaging Sciences, Amgen, Thousand Oaks, California, United States of America; 4 Center for Medical Image Science and Visualization (CMIV), Department of Medical and Health Sciences, Linköping University, Linköping, Sweden; 5 Wolfram MathCore, Linköping, Sweden; 6 National Center for Toxicological Research, Division of Neurotoxicology, United States Food and Drug Administration, Jefferson, Arkansas, United States of America; 7 Personalised Healthcare and Biomarkers, Imaging group, Innovative Medicines and Early Development Biotech Unit, AstraZeneca, Gothenburg, Sweden; 8 Biostatistics, Quantitative Biology, Discovery Sciences, Innovative Medicines and Early Development, AstraZeneca R&D, Cambridge, United Kingdom; 9 Safety Assessment, GlaxoSmithKline, Research Triangle Park, Durham, North Carolina, United States of America; 10 National Center for Toxicological Research, Division of Systems Biology, United States Food and Drug Administration, Jefferson, Arkansas, United States of America; 11 Safety and ADME Translational Sciences, Drug Safety and Metabolism, AstraZeneca, Cambridge, United Kingdom; 12 Department of Comparative Biology and Safety Sciences, Amgen Inc., Thousand Oaks, California, United States of America; 13 Division of Pharmacotherapy and Experimental Therapeutics, UNC Eshelman School of Pharmacy, University of N orth Carolina at Chapel Hill, Chapel Hill, North Carolina, United States of America; 14 Antaros Medical, BioVenture Hub, Mölndal, Sweden; 15 MedTech West, Chalmers University of Technology, Gothenburg, Sweden; Henry Ford Health System, UNITED STATES

## Abstract

Drug-induced liver injury (DILI) is a leading cause of acute liver failure and transplantation. DILI can be the result of impaired hepatobiliary transporters, with altered bile formation, flow, and subsequent cholestasis. We used gadoxetate dynamic contrast-enhanced magnetic resonance imaging (DCE-MRI), combined with pharmacokinetic modelling, to measure hepatobiliary transporter function *in vivo* in rats. The sensitivity and robustness of the method was tested by evaluating the effect of a clinical dose of the antibiotic rifampicin in four different preclinical imaging centers. The mean gadoxetate uptake rate constant for the vehicle groups at all centers was 39.3 +/- 3.4 s^-1^ (*n* = 23) and 11.7 +/- 1.3 s^-1^ (*n* = 20) for the rifampicin groups. The mean gadoxetate efflux rate constant for the vehicle groups was 1.53 +/- 0.08 s^-1^ (*n* = 23) and for the rifampicin treated groups was 0.94 +/- 0.08 s^-1^ (*n* = 20). Both the uptake and excretion transporters of gadoxetate were statistically significantly inhibited by the clinical dose of rifampicin at all centers and the size of this treatment group effect was consistent across the centers. Gadoxetate is a clinically approved MRI contrast agent, so this method is readily transferable to the clinic. *Conclusion*: Rate constants of gadoxetate uptake and excretion are sensitive and robust biomarkers to detect early changes in hepatobiliary transporter function *in vivo* in rats prior to established biomarkers of liver toxicity.

## Introduction

Drug-induced liver injury (DILI) is a leading cause of acute liver failure and transplantation [1]. DILI is a major challenge for the pharmaceutical industry and regulatory agencies. Twenty-nine percent of drugs withdrawn from the market between 1998 and 2008 were withdrawn due to liver toxicity [2]. The costs of additional clinical trials and delays in reaching the market can be substantial [3]. The ‘classic’ biomarker of liver injury, serum alanine aminotransferase (ALT), is not a valid biomarker of the severity of liver injury and is not a test of liver function [4]. An elevation in serum total bilirubin is highly specific for liver disease, but often occurs late in the disease process [4]. Ideally, more sensitive biomarkers that respond early, prior to irreversible injury, would offer improved outcomes for patients [5].

Pharmaceutical companies reduce the likelihood that new drugs will induce limiting toxicities by identifying hazards early in the research phase of drug development. *In vitro* approaches, such as membrane vesicle assays, transporter over-expressing cell systems, and sandwich-cultured hepatocytes, are used to reduce the likelihood that candidate drugs selected for clinical development will cause DILI in humans [6]. These approaches have improved the specificity of DILI predictions to 90–95%; however, the sensitivity of these screens is still only 50% [7]. Regulatory animal toxicity studies also predict only 55% of drugs that induce DILI [8]. The importance of hepatic transporters and examples of how they may affect hepatocyte drug concentrations has been reviewed [9, 10]. Inhibition of hepatobiliary efflux transporters may lead to changes in drug exposure in the hepatocyte and consequent hepatotoxicity with only minor changes in drug plasma exposure [11]. New tools, translatable to the clinic, need to be developed to measure the function of efflux transporters.

Hepatobiliary MRI contrast agents such as gadoxetate dimeglumine (Gd-EOB-DTPA, Eovist^®^; Primovist^®^; Bayer HealthCare Pharmaceuticals, Berlin, Germany) and gadobenate dimeglumine (Gd-BOPTA, MultiHance; Bracco Imaging, Milan, Italy) are used in routine medical practice to detect focal liver tumors [12, 13]. Gadoxetate is taken up by hepatocytes *via* organic anion-transporting polypeptide 1 (Oatp1) transporters and therefore not taken up by tumor cells. It is pumped into the bile by multidrug resistance-associated protein 2 (Mrp2) transporters [14–16]. In addition to detecting liver tumors, gadoxetate has been used to monitor hepatobiliary transporter activity in preclinical [17–20] and clinical MRI studies [21, 22]. These studies have used empirical data models to measure uptake of gadoxetate by hepatocytes. Huppertz et al. [23] investigated the effect of a clinical dose of erythromycin on the relative enhancement of a liver contrast agent, but did not see a statistically significant effect. The signal intensity in DCE-MRI reflects the changes in contrast agent concentration after a bolus injection. Gadoxetate DCE-MRI with a unique kinetic analysis to describe intracellular uptake and excretion has been used to examine inhibition of hepatobiliary transporters at toxicological doses of a drug in one animal study [24] and in one clinical study of patients with hepatobiliary disease [25]. In this pharmacokinetic model, gadoxetate uptake was associated with the function of the Oatp1 transporter and efflux was associated with Mrp2 function.

It is not known whether gadoxetate DCE-MRI can detect the effect of a clinical dose of a marketed drug on hepatobiliary transporter function in rats. In addition, the need for multicenter trials to evaluate novel biomarkers is recognized in the clinical setting [26], however, these studies are rarely conducted for preclinical biomarkers [27]. The aim of the present study was to evaluate liver transporter function with gadoxetate DCE-MRI in rats treated with either vehicle or a clinical dose of rifampicin, at four different preclinical imaging centers (Amgen, AstraZeneca, U.S. Food and Drug Administration (FDA) and GlaxoSmithKline) using various MR scanner field strengths. Rifampicin is an antibiotic used to treat tuberculosis and is known to inhibit the hepatobiliary transporters Oatp1 and Mrp2 [28].

The present work was conducted as part of the International Life Sciences Institute Health and Environmental Sciences Institute Translational Imaging Committee program. This is a consortium of industry, government and academic scientists whose mission is to advance development of biomarkers of target organ toxicity that bridge from the preclinical to the clinical stages of drug development. Participants in this consortium subgroup, which focused on liver imaging, were Amgen, AstraZeneca, FDA, GlaxoSmithKline, and The University of North Carolina at Chapel Hill.

## Materials and methods

### Compound selection

A literature search found 97 marketed compounds that inhibited the hepatic uptake transporter Oatp1 and efflux transporter Mrp2. Compounds with missing or inconsistent IC_50_ values, IC_50_ values well above the reported maximum plasma concentration, or where there could be a perceived conflict of interest, were removed, leaving a shortlist of five compounds, cyclosporine A, diclofenac, fusidate, ketoprofen and rifampicin. Rifampicin was selected as the model inhibitor for this study based on the relative IC_50_ values of Oatp1 and Mrp2, which allowed both the inhibitor drug and contrast agent to enter the hepatocytes.

### Animal experiments

All experiments were conducted in accordance with guidelines and laws for the care and use of laboratory animals. Experiments conducted at Amgen (Thousand Oaks, CA), GlaxoSmithKline (King of Prussia, PA) and FDA (Jefferson, AR) were approved by the local Institutional Animal Care and Use Committees. Experiments conducted at AstraZeneca (Gothenburg, Sweden) were approved by the regional Ethical Committee on Animal Experiments.

Male Wistar Han rats (Charles River, Raleigh, NC; Wilmington, MA; and Sulzfeld, Germany) weighing 250–300 g were used in the study. Each center purchased rats from the local Charles River supplier. Animals were housed in rooms with temperature regulated to 20±2 °C and a 12/12 hour light/dark cycle with artificial light. Rats were acclimatized for at least one week prior to the study. Animals were allowed access to food and water *ad libitum* as well as enrichment. Each center purchased standard rat chow from its local supplier. Rats were not fasted and all experiments were run during light cycle.

Rats were randomly assigned into rifampicin or vehicle treated groups. Rats were anesthetized with 2.5% isoflurane in air and anesthesia was maintained at 1.8–2.0% throughout the procedure. The tail vein was cannulated with a 26G catheter. Rectal temperature and respiration rate were monitored throughout the imaging procedure (SA instrument, New York, USA) and maintained at 35–37 °C and 50–60 breaths/min.

Rats were sacrificed without regaining consciousness by an overdose of isoflurane and terminal cardiac puncture at the end of the experiment.

### Sample size justification

The intraclass correlation coefficient (ICC) measure of agreement between centers was unknown, and different animals were used at each center. Hence, the sample size was based on detecting a difference between treatments in a multicenter study. Using standard equations for cluster trials [29], if six rats/group were used with four centers (*i*.*e*., a total sample size of 48), even with an ICC up to 0.95 the effective sample size was still above that required for the equivalent four rats/group single-site study. This sample size was chosen for pragmatic reasons to power the study for a wide range of ICC values, and to allow for attrition.

### Formulation, administration and dosing of compounds

Rifampicin (Sigma-Aldrich, Munich, Germany) was formulated at 5 mg/mL in 5% DMSO, 10% PEG 300, 20% cavitron (hydroxypropyl-beta-cyclodextrin) in water while the control group received the vehicle alone. A commercialized solution of gadoxetate (Eovist^®^, Primovist^®^ Bayer HealthCare Pharmaceuticals, Berlin, Germany) was used at the clinically recommended concentration of 0.25 mmol/mL.

### MRI data acquisition

MR images were acquired at various field-strengths (one 4.7 T, one 7.0 T and two 9.4 T magnets; Bruker, Billerica, MA). Scout images were acquired with IntraGateFLASH (repetition time 36–88 ms, echo time 1.2–1.8 ms, flip angle 30°, matrix 128x128, Field-of-View 60x60 mm^2^, 10 slices, and slice thickness of 2 mm). A coronal slice orientation intersecting both the liver and the spleen was selected for the DCE-MRI sequence. DCE-MRI images were acquired with a single-slice T1-weighted gradient-echo sequence (IntraGateFLASH, repetition/echo time 7.1/1.8 ms, flip angle 30°, matrix 256x256 or 128x128), Field of view 60x60 mm^2^, slice thickness of 2 mm) over 60 minutes. IntraGateFLASH is a retrospective triggering technique which was set to accept data from the quiescent 70% of the respiratory cycle [30]. Cardiac triggering was not used. DCE-MRI imaging started 5 minutes prior to IV administration of 25 μmol/kg gadoxetate through the tail vein catheter with an automatic microinjection pump at 250 μl/min. Images were reconstructed with a temporal resolution of 1 minute.

### Study 1: Rifampicin dose response

In a pilot single-center study, rats received a single IV injection of rifampicin at 1 mg/kg (n = 3), 3 mg/kg (n = 3), 6 mg/kg (n = 4) or 20 mg/kg (n = 3) while the vehicle group (n = 8) received the vehicle alone. DCE-MRI acquisition commenced 60 minutes after rifampicin or vehicle injection and continued for another 60 minutes.

### Study 2: Rifampicin pretreatment evaluation

In a single-center pilot study, rats received 10 mg/kg IV rifampicin at 15 (n = 2), 30 (n = 2) or 60 minutes (n = 2) before the DCE-MRI acquisition. The control group (n = 3) received vehicle alone 60 minutes before the DCE-MRI acquisition.

### Study 3: Repeatability of the DCE-MRI assay

In a multi-center study in four centers, each center included six vehicle treated rats and six rats treated with 10 mg/kg rifampicin (total 48 rats studied). Results from individual centers (Amgen, AstraZeneca, FDA and GlaxoSmithKline) are presented anonymized as i, ii, iii and iv. Each rat received an IV injection of rifampicin or the vehicle 60 minutes prior to the DCE-MRI acquisition.

### Image analysis

The MR signal was assessed in regions-of-interests, manually drawn by each site on their own images, covering the liver and the spleen using the image processing software ImageJ (v1.47, National Institute of Health, USA), ParaVision (Bruker, Billerica, MA), or Analyze 11.0 (AnalyzeDirect, Overland Park, KS) depending on the center. The area under the curve (AUC) was calculated as the sum of the relative enhancement (RE) at each time-point during DCE-MRI. RE at a given time-point is defined as:

$${RE}_{t}\;=\;\left({SI}_{t}-{SI}_{0}\right)/{SI}_{0}\times 100\%$$ (1)

where SI_0_ is the average pre-contrast MR signal intensity and SI_t_ is the MR signal intensity at time *t*.

Animals were excluded from the kinetic analysis when the images did not cover the spleen (one rat in center i, one rat in center ii and one rat in center iii). Spleen data is essential for the pharmacokinetic analysis described in the next section.

### Pharmacokinetic model

Gadoxetate concentrations were estimated from the liver and spleen DCE-MRI as described by Ulloa et al. [24] using the literature values for the pre-contrast longitudinal relaxation rate, and post-contrast longitudinal relaxation calculated from the IntraGate FLASH images using the signal equation of a saturation recovery spoiled gradient echo. Contrast agent kinetics were assumed to be well-approximated by a linearized expression (Eq 2) of the model given by Ulloa et al. [24] where *k*_*1*_ describes the uptake rate of gadoxetate from the extracellular space to the hepatocytes, *k*_*2*_ describes the efflux rate of gadoxetate from the hepatocytes [25], and the input function is provided by the spleen data.

$$\frac{dC_{hep}(t)}{dt}\;=\;k_{1}C_{ES}(t)-k_{2}C_{hep}(t)$$ (2)

*C*_*hep*_(*t*) and *C*_*ES*_(*t*) are the gadoxetate concentrations in hepatocytes and extracellular space, respectively. The volume fractions of the extracellular spaces in liver and spleen also were taken from Ulloa et al. [24].

Precontrast longitudinal relaxation times (T1) in the liver and the spleen, and gadoxetate relaxivities (r1) were estimated from literature data [31–36] and are summarized in Table 1. A gamma-variate curve was fitted to the spleen data to reduce the influence of noise. For each study subject, *k*_1_ and *k*_2_ were estimated using a nonlinear global optimization algorithm [37] implemented in Mathematica (v10.0.2.0, Wolfram Research Inc., Champaign, IL) [25]. Pharmacokinetic modeling of data from all sites was conducted by an independent third party (Wolfram MathCore, Linköping, Sweden).

**Table 1:** Estimated values for relaxation time and relaxivity at the employed field strengths.

B_0_ [T]	Liver T_1_ [ms]	Spleen T_1_ [ms]	r_1_ [M^-1^ms^-1^]
4.7	900	1200	5.5
7.0	1050	1370	5.1
9.4	1180	1510	4.8

Liver and spleen T_1_s at the employed field strengths were found *via* curve fitting to literature data [31–36]. T_1_s were assumed to follow the form T_1_ = A*v*B, where A and B are constants and *v* the ^1^H resonance frequency. Gadoxetate relaxivities also were estimated *via* curve fitting based on references [38, 39].

### Plasma chemistry analysis

Plasma biomarkers of liver injury were measured at a single site during Study 3. Baseline blood samples were obtained from the tail vein through a catheter immediately before rifampicin dosing. Endpoint samples were obtained by cardiac puncture. Blood was collected in EDTA and heparin tubes and centrifuged at 1700 rpm for 10 minutes at 4 °C. Plasma was stored at -80 °C. Alanine aminotransferase, aspartate aminotransferase, alkaline phosphatase, and total and direct bilirubin were measured in plasma samples on a Horiba Pentra 400 Clinical Chemistry instrument.

### Plasma miRNA-122

Total miRNA was extracted from plasma samples using miRNeasy MiniKit (Qiagen^®^, Hilden, Germany). cDNA was synthesized from the extracted miRNA using a TaqMan^®^ MicroRNA Reverse Transcription kit (Life Technologies^®^, Carlsbad, CA). qPCR to amplify miRNA-122 and the reference gene cel-39 were run using TaqMan Fast Universal PCR Master Mix and the primers has-miR122 and cel-miR-39 (Life Technologies^®^, Carlsbad, CA). qPCR was run on a QuantStudio7 Flex (Life Technologies^®^, Carlsbad, CA) device.

### Statistical analysis

Unless otherwise stated, all values are reported as the mean +/- SEM. Unpaired *t*-tests and statistical analyses were performed using GraphPad Prism 6.00 (La Jolla, CA) on maximum RE values, plasma chemistry and miRNA122 copy number. Results were considered to be significant for a p value <0.05 (*), p<0.01 (**), p<0.001 (***) and p<0.0001 (****).

A mixed effects model was fitted to data from Study 3, where treatment and center were treated as fixed effects and animals were random effects. The main model effects were treatment, center, and the treatment-by-center interaction. Results were considered to be significant for a p value <0.05 (*), p<0.01 (**), p<0.001 (***) and p<0.0001 (****). Variance components were extracted to compare sources of variation, such as that caused by center. ICC, a measure of the proportion of variation explained within each center, was estimated using SAS 9.2 (SAS Institute, Cary, NC).

The sample size for a range of treatment effects in both single and multicenter settings was estimated using nQuery software (Statistical Solutions^LTD^, Cork, Ireland). The standardised effect size is defined as the mean for vehicle rats at all centers minus the mean for rifampicin treated rats at all centers, divided by the global standard deviation in all centers.

## Results

**Fig 1:**
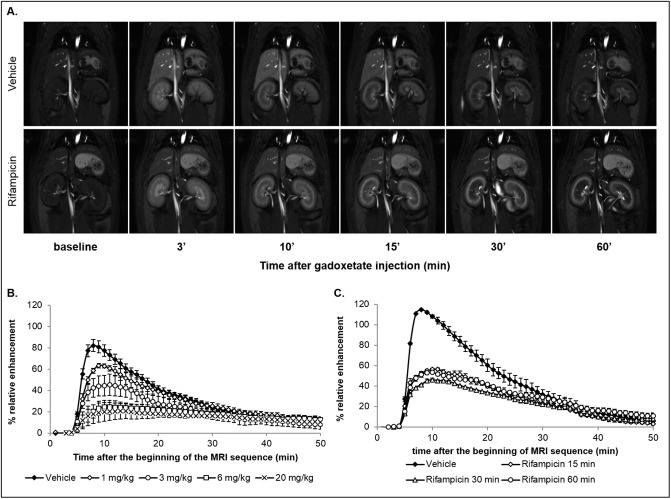
MR images and RE time curves from studies 1 and 2. Typical vehicle treated and 10 mg/kg rifampicin treated rats pre- and post- injection of gadoxetate (25 μmol/kg) (A); DCE-MRI RE curves after 1 mg/kg (n = 3), 3 mg/kg (n = 3), 6 mg/kg (n = 4) or 20 mg/kg (n = 3) of rifampicin or vehicle (n = 8) dosed IV (B); DCE-MRI RE curves in vehicle treated rats (n = 3) or 15 minutes (n = 2), 30 minutes (n = 2) or 60 minutes (n = 2) after dosing with 10 mg/kg rifampicin (C). Values are presented as mean +/- SEM.

A MR signal peak was generally observed 3 minutes after the injection of gadoxetate (Fig 1A). Greater RE of MR signal intensity was observed in the liver of rats receiving the vehicle alone compared to the rats treated with rifampicin.

### Study 1: Rifampicin dose response

Rifampicin treatment decreased the maximum mean RE in the liver from 82% in the vehicle group, to 63% in the 1 mg/kg group, 45% in the 3 mg/kg group, 25% in the 6 mg/kg group and 21% in the 20 mg/kg group (Fig 1B). Effects were significant for doses 3 to 20 mg/kg (*p* <0.01). The decrease in the RE compared to the vehicle group was proportional to the dose of inhibitor administered between 1 mg/kg and 6 mg/kg, while the decrease in RE was similar between 6 mg/kg and 20 mg/kg rifampicin. Based on these results, the clinical dose of 10 mg/kg rifampicin was chosen for subsequent studies.

### Study 2: Rifampicin pretreatment time evaluation

The impact of time between treatment with rifampicin and liver transporter inhibition was evaluated in rats (Fig 1C). A statistically significant (p <0.0001) decrease in maximum RE was observed after 15-, 30- or 60-minutes treatment with 10 mg/kg rifampicin compared to the control group (maximum RE of 115%). A similar reduction in maximum RE of between 46% and 57% was observed regardless of pretreatment time (Fig 1C). 60 minutes pretreatment with rifampicin was selected for the multicenter study.

### Study 3: Multicenter DCE-MRI assay

**Fig 2:**
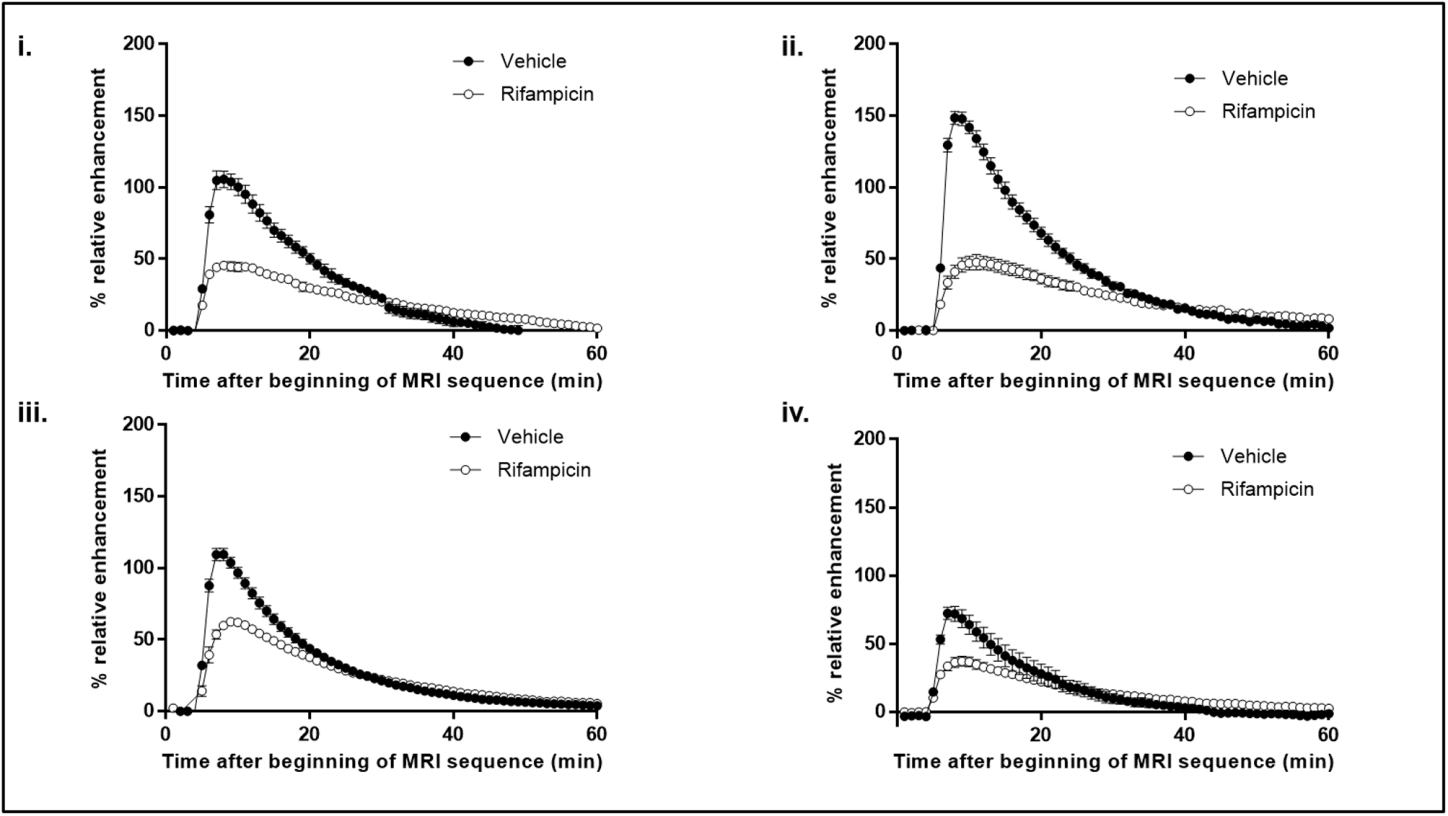
RE time curves from the 4 centers in the multicenter study. Gadoxetate (25 μmol/kg) DCE-MRI RE curves 60 minutes after IV dosing with vehicle (*n* = 6 per center) or 10 mg/kg rifampicin (*n* = 6 per center). Images were acquired at four independent research centers i, ii, iii, and iv. Results are presented as mean +/- SEM.

The robustness of the method was evaluated by running the gadoxetate DCE-MRI assay 60 minutes after pretreatment with 10 mg/kg rifampicin at four independent research centers (Fig 2).

The maximum RE in the vehicle groups varied between sites from a minimum of 75% (Fig 2iv) to a maximum of 150% (Fig 2ii) and these differences were statistically significant (*p* <0.0001). Rifampicin caused a statistically significant decrease in maximum mean RE compared to vehicle at all centers (*p* <0.0001). The treatment-by-center interaction term was significant (*p* <0.0001) indicating that the treatment effect varied between centers (Table 2). The ICC was 0.82 indicating that much of total variance was between centers.

**Table 2:** Mixed model results on *k*_*1*_, *k*_*2*,_ AUC and maximum RE parameters from multicenter *in vivo* Study 3.

	Parameter	F	DF	Estimate	SE	LCL	UCL	p-value
*k1* (s^-1^)	Group	89.9	35					<0.0001
	Center	10.3	35					<0.0001
	Interaction	2.27	35					0.098
	Rifampicin-Vehicle			-27.52	2.90	33.42	21.63	<0.0001
*k2* (s^-1^)	Group	37.8	35					<0.0001
	Center	6.5	35					0.001
	Interaction	0.74	35					0.53
	Rifampicin-Vehicle			-0.58	0.10	-0.78	-0.39	<0.0001
AUC (RE.min)	Group	35.7	39					<0.0001
	Center	23.0	39					<0.0001
	Interaction	5.72	39					0.0024
	Rifampicin-Vehicle			-5.14	0.86	-6.88	-3.40	<.0001
Maximum RE	Group	434	35					<0.0001
	Center	38.6	35					<0.0001
	Interaction	22.7	35					<0.0001
	Rifampicin-Vehicle			-0.61	0.03	-0.67	-0.55	<0.0001

F: F-distribution; DF: Degrees of Freedom of a distribution; SE: Standard Error: LCL: Lower Confidence Level (of a 95% Confidence Interval CI); UCL: Upper Confidence Level

Results were similar for AUC. The treatment-by-center interaction term was also significant (*p* <0.01), indicating the treatment effect varied between centers. The ICC was 0.47.

**Fig 3:**
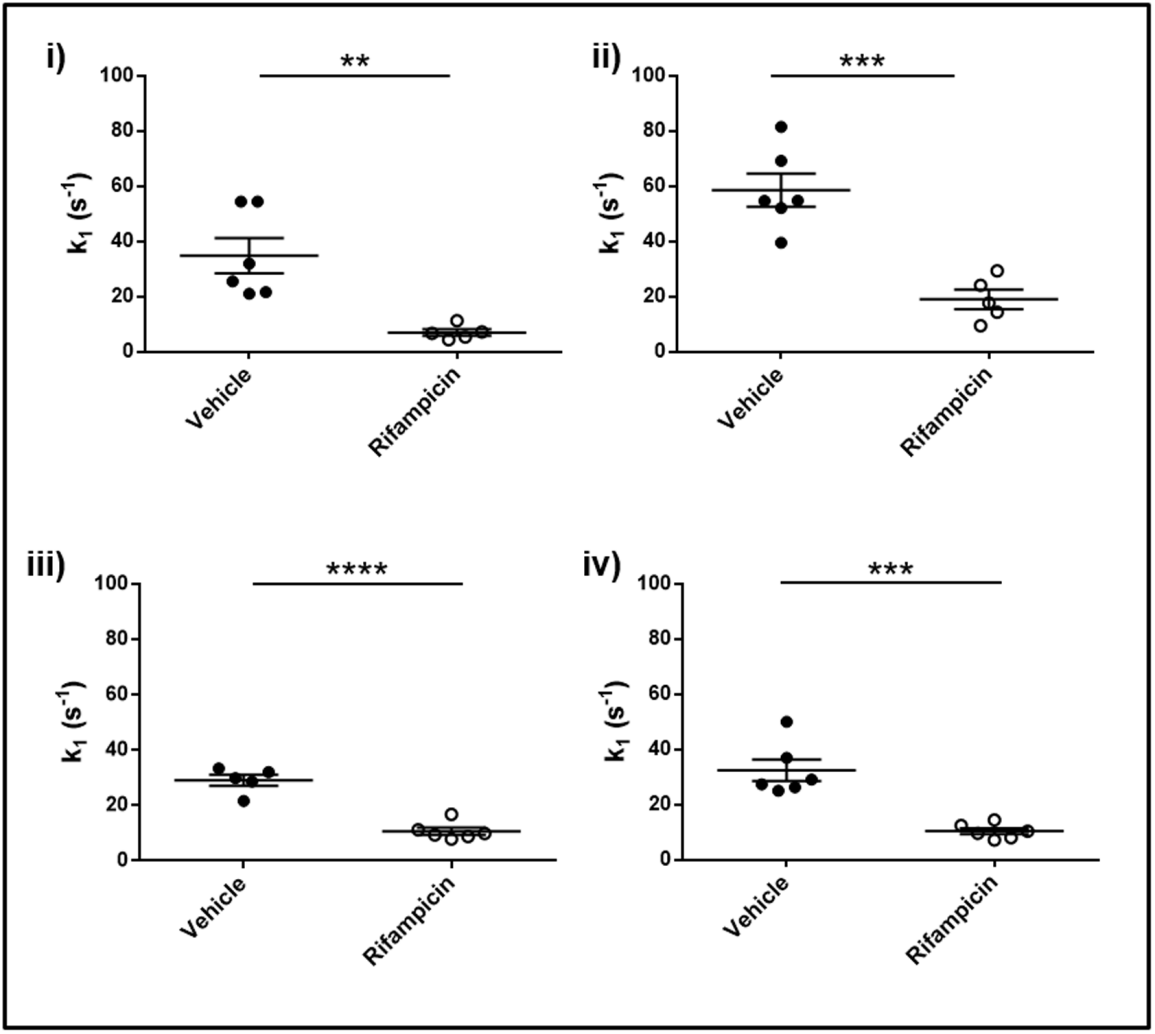
Gadoxetate influx parameters in the multicenter study. Pharmacokinetic parameters describing the impact of rifampicin on gadoxetate uptake (*k*_*1*_) at each of the four research centers: i, ii, iii, and iv. In each center, 6 animals are vehicle treated (dark circles) while 6 animals are rifampicin treated (open circles). Results are presented as mean +/- SEM.

*k*_*1*_ (gadoxetate uptake rate constant) for the vehicle group was 39.3 +/- 3.4 s^-1^ (*n* = 23) and 11.7 +/- 1.3 s^- 1^ (*n* = 20) for the rifampicin treated group S1 Table. There were significant differences in the values for vehicle observed at the four centers (*p* <0.0001), however all centers detected a significant decrease (*p* <0.001) in *k*_*1*_ in the rifampicin treated group compared to the vehicle treated group (Fig 3). The overall treatment effect for rifampicin vehicle was -27.5 s^-1^. The *k*_*1*_ treatment-by-center interaction term was non-significant (*p* = 0.10), indicating that there was no evidence against the same relative treatment effect at all centers. Both the treatment and center effects for *k*_*1*_ were highly significant (*p* <0.0001). All key comparisons are presented in Table 2. Although there was an imbalance in the sample sizes between centers, this had a negligible influence on the original sample size assumptions. The ICC for treatment effect, measuring relative clustering between centers, was 0.35, indicating that much of the variance comes on the individual animal level.

**Fig 4:**
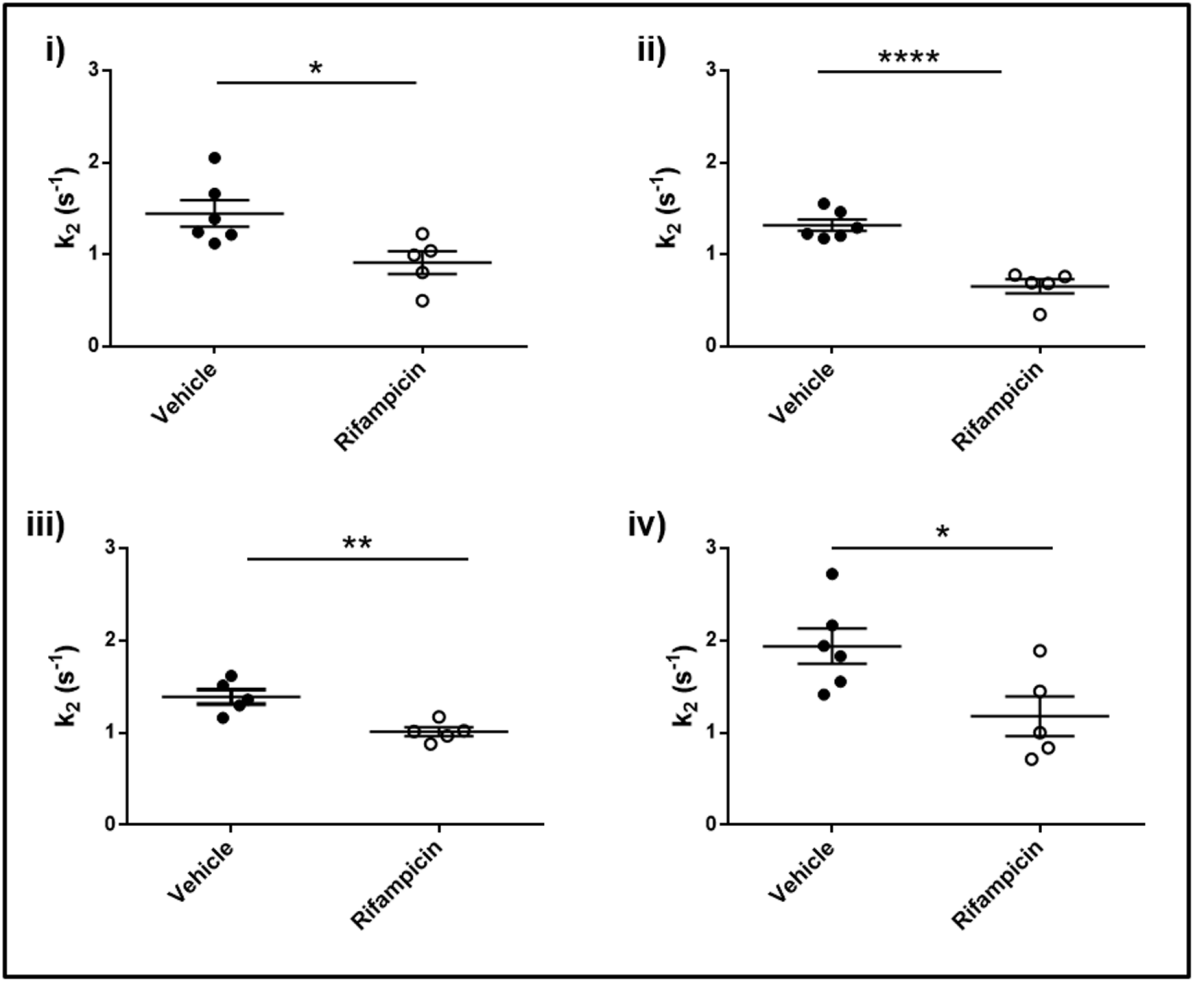
Gadoxetate efflux parameters in the multicenter study. Pharmacokinetic parameters describing the impact of rifampicin on gadoxetate efflux (*k*_*2*_) at each of the four research centers: i, ii, iii, and iv. In each center, 6 animals are vehicle treated (dark circles) while 6 animals are rifampicin treated (open circles). Results are presented as mean +/- SEM.

Similarly, for *k*_*2*_ (gadoxetate efflux rate constant) the mean for the vehicle treated group was 1.53 +/- 0.08 s^-1^ (*n* = 23) and the mean for the rifampicin treated group was 0.94 +/- 0.08 s^-1^ (*n* = 20). There were significant differences in the values for vehicle observed at the four centers and all centers detected a significant decrease with treatment (*p* = 0.001) (Fig 4). The overall treatment effect size was -0.58 s^-1^. The treatment-by-center interaction term was non-significant (*p* = 0.53), again indicating that there was no evidence against the same relative treatment effect at all centers. The ICC was 0.02, which was the lowest ICC value of all the endpoints and showed that between-center variability had the least effect.

**Fig 5:**
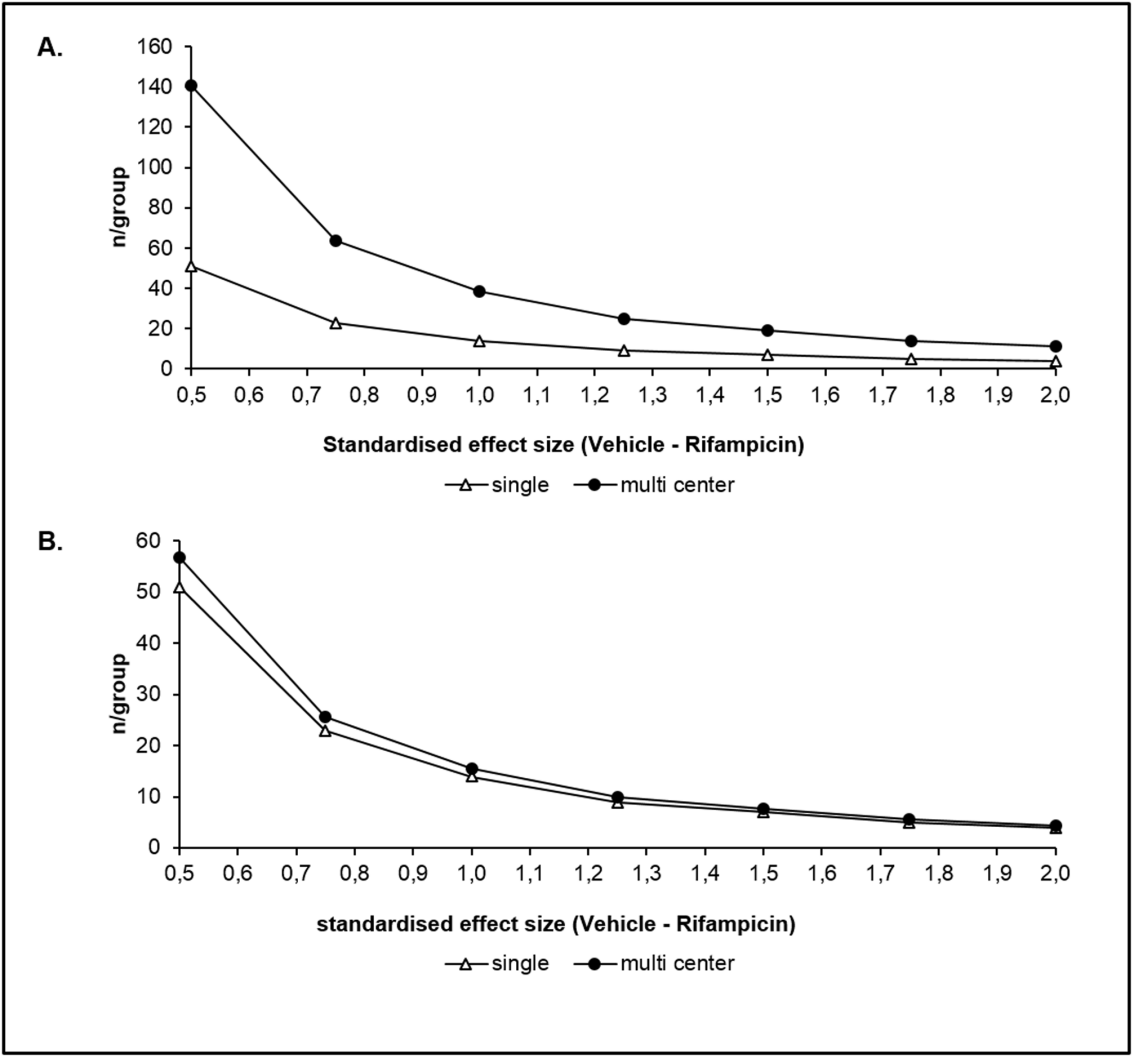
Sample size calculations for single- and multi-center studies. *k*_*1*_ (A) and *k*_*2*_ (B) in single-center (open triangles) or multi-center (closed circles) studies. The number of animals per center (*m*) is set to 6 in this example. *n* is the total number of animals needed to achieve statistical significance for a given standardized effect size.

Pooled estimates of variability for the vehicle and rifampicin treated groups for *k*_*1*_ (SD = 12.2 and 4.3 s^-1^, respectively) and *k*_*2*_ (SD = 0.32 and 0.30 s^-1^, respectively) were estimated by taking a weighted average of the variance for each treatment from the four centers. Based on a two-group Satterthwaite *t*-test for unequal variances, for a 1-sided test with 80% power and alpha of 5%, power curves for single-center studies were generated. Using the ICC value to calculate the design expansion (DE) factor for a given number of animals per center (m), the sample sizes for multi-center studies were also generated [29] (Fig 5).

### Plasma chemistry and miRNA-122 copy number

**Fig 6:**
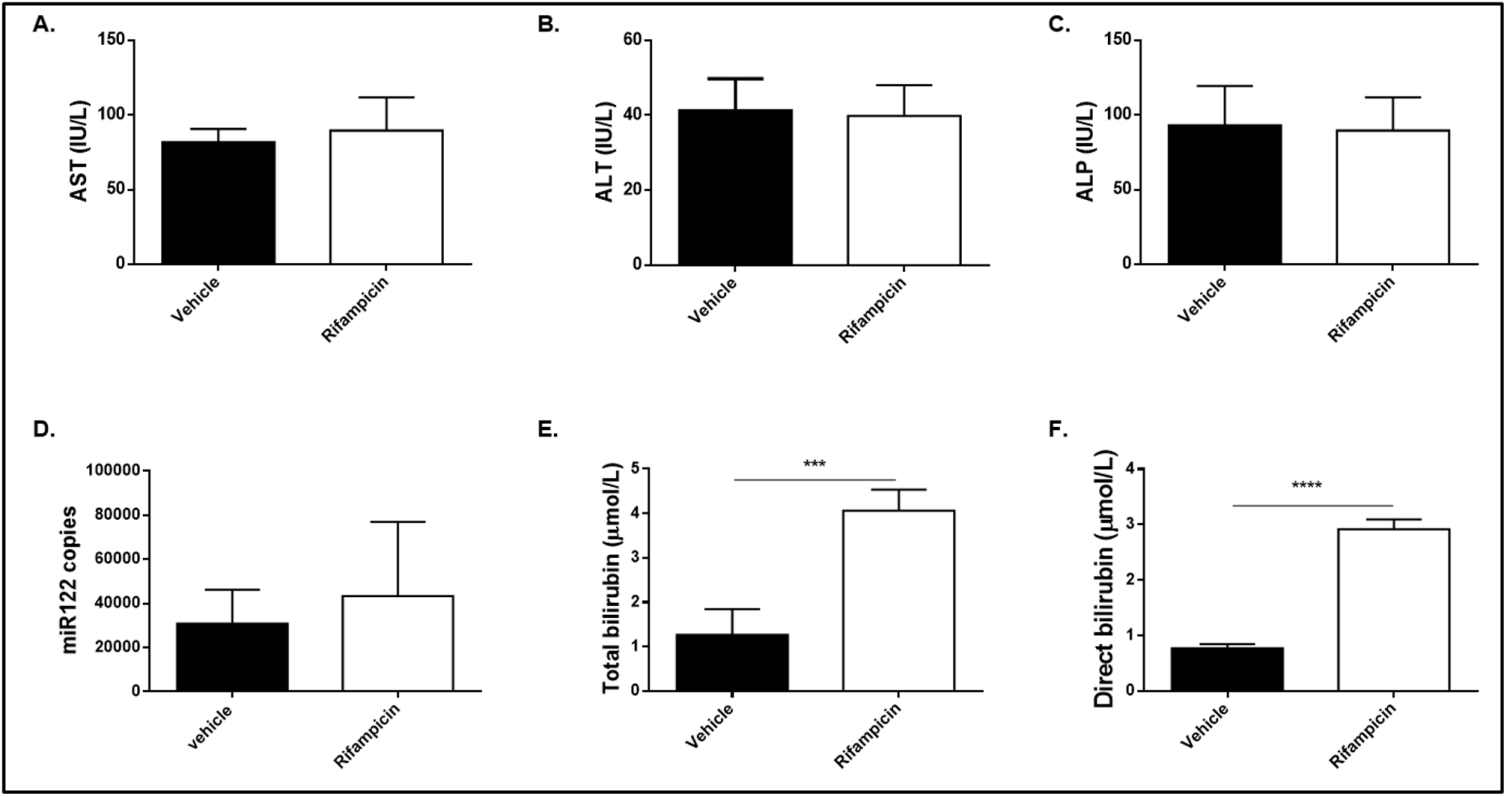
Plasma biomarkers of liver injury. Plasma biomarkers of liver injury 2 hours after treatment with either vehicle (n = 5, dark bars) or 10 mg/kg rifampicin (n = 6, light bars). Routine hepatic enzyme biomarkers aspartate aminotransferase (AST) (A), alanine aminotransferase (ALT) (B) and alkaline phosphatase (ALP) (C) were measured as well as the early biomarker of liver injury miR-122 (D). Total (E) and direct (F) bilirubin were measured as a biomarker of Mrp2 inhibition. Direct bilirubin was measured in plasma samples collected two hours after treatment with rifampicin. Results are presented as mean +/- SEM. Differences between groups were evaluated using Student’s *t*-test with p<0.5 (*), p<0.01 (**), p<0.001 (***) and p<0.0001 (****).

Evidence of potential for induction of liver toxicity was evaluated using plasma biomarkers (Fig 6). There was no significant difference in any of the measured hepatic enzymes aspartate aminotransferase, alanine aminotransferase or alkaline phosphatase between the rifampicin and vehicle treated groups (Fig 6A, 6B and 6C) with these plasma biomarkers of potential DILI. Furthermore, there was no significant difference between miRNA-122 copy number in plasma samples between groups (Fig 6D).

A significant increase (*p* <0.0001) in total and direct bilirubin levels 2 hours after the rifampicin treatment was observed (Fig 6E and 6F).

## Discussion

Rifampicin exhibited a dose-dependent impact on the relative enhancement of MR signal after gadoxetate injection. Inhibition of gadoxetate transport in the rat liver was detected at one-third of the 10 mg/kg clinical dose of rifampicin, indicating that the assay has the sensitivity to detect effects on hepatobiliary transporter function in rats at clinically relevant doses. The response was not sensitive to the duration between rifampicin dosing and the start of DCE-MRI in the range of 15 to 60 minutes.

The robustness of the gadoxetate DCE-MRI assay was tested by conducting the same experiment in four different laboratories using MR scanners at three different field strengths and Han Wistar rats from local suppliers. Each center measured MR signal intensity in liver and spleen in their own images. Results from all sites were sent to a central lab for pharmacokinetic analysis. The mixed effects model showed that the center effect was statistically significant for *k*_*1*,_ *k*_*2*_, maximum RE and AUC, indicating significant differences between sites. These differences may reflect differences in the rats from the local suppliers, the *in vivo* procedures, the MRI scanners, or differences in the way the regions of interest were drawn.

The mixed effects model also showed a statistically significant treatment effect for *k*_*1*,_ *k*_*2*_, maximum RE and AUC. The size of this treatment group effect was consistent for *k*_*1*_ and *k*_*2*_ across the centers, but for AUC and maximum RE the size varied by center, which suggests *k*_*1*_ and *k*_*2*_ produce more robust results. This indicates that a similar drug effect can be detected independent of test center when using *k*_*1*_ and *k*_*2*_ as endpoints. The descriptive analysis techniques, maximum RE and AUC, have good signal-to-noise ratio and low variance at individual sites, however the effect size is not reproducible between sites, potentially reflecting a greater dependence on experimental parameters. Power curves are presented that will allow other investigators to appropriately power their own experiments given a predetermined effect size and assuming similar standard deviations to Study 3.

Neither the established biomarkers of liver injury, aspartate aminotransferase, alanine aminotransferase and alkaline phosphatase, nor the novel biomarker miRNA-122, showed a difference between rifampicin and vehicle treated groups two hours after dosing with rifampicin. This confirms the results of Lenhard et al. where changes in transporter function were observed without an increase in liver injury enzymes [19]. In contrast, both total and direct bilirubin were elevated. These results are consistent with Watanabe et al. [40], who demonstrated that an increase in total and direct bilirubin is a biomarker of hepatobiliary transporter inhibition. Rifampicin toxicity is only observed at much higher doses [41], emphasizing that gadoxetate DCE-MRI detects hepatobiliary transporter function and not downstream effects of liver toxicity.

Gadoxetate is taken up by hepatocytes *via* Oatp1 transporters and is pumped into the bile by Mrp2 transporters [14, 15]. Jia et al. showed that gadoxetate clearance from the liver is delayed in Mrp2-deficient rats and that no gadoxetate could be detected in feces, indicating transport into bile is blocked [15]. Based on this data, it is unlikely that other transporters, such as Bsep (Bile Salt Export Pump), transport gadoxetate across the canalicular membrane. Thus, in the pharmacokinetic model used in the present study, Oatp1 function was associated with *k*_*1*_ (uptake) and Mrp2 function with *k*_*2*_ (efflux). There may be transport of gadoxetate back into plasma due to basolateral transporters such as multidrug resistance-associated protein 3 (Mrp3) [42]. Mrp3 is induced by liver disease or drugs, and therefore in the healthy animals used in the present study we assumed that most efflux occurs *via* the canalicular membrane. Compared to descriptive analysis techniques such as RE and AUC, kinetic models have a physical meaning and can give insight into the mechanism of drug action [43, 44]. Specifically, the model employed in the present study can separate the impact of a drug on uptake and efflux rates.

Before novel biomarkers are applied in drug development, they undergo a method validation [45, 46]. The validity of imaging biomarkers depends on the use of medical imaging techniques and images analysis [47, 48]. Despite widespread recognition of the importance of multicenter studies in biomarker validation, to the best of our knowledge, the current study is the first multicenter preclinical imaging study. The four imaging centers agreed on a common imaging protocol, animal species and strain, test substance and dose, and that all pharmacokinetic modelling would be done by an independent third party. However, there is no guarantee that animals from various local suppliers are identical, so variation will be higher than for a more controlled population at a single site. In addition, there were different MRI scanners at each site with different magnetic field strengths, gradient coils, and hardware releases. This multi-center study has shown that comparable drug effect size can be found in different laboratories when using pharmacokinetic endpoints to measure gadoxetate uptake and excretion. This result gives confidence that further consortium work to evaluate gadoxetate DCE-MRI can be reproduced between multiple laboratories. For the individual pharmaceutical companies, it gives confidence that comparable results may be obtained even if a gadoxetate DCE-MRI study is outsourced due to lack of internal resources. Results based on descriptive analysis techniques should be avoided in the multi-center setting.

To qualify this biomarker for use in drug development, further work needs to be conducted to explore effect size, robustness and timing with a broad range of drugs that alter transporter function and induce adaptation or DILI to understand the link between inhibition of hepatobiliary transporters in preclinical studies and the development of DILI in the clinic. MRI is directly translatable between animal studies and the clinic [49], and gadoxetate is a contrast agent approved for IV use to detect and characterize focal liver lesions in patients. Gadoxetate DCE-MRI detects changes in transporter activity due to polymorphisms in healthy subjects [50], and can predict hyperbilirubinemia during treatment of chronic hepatitis C patients [51]. Further studies should be conducted to determine whether gadoxetate DCE-MRI can detect drug-induced inhibition of hepatobiliary transporters in clinical studies. The combination of preclinical and clinical studies with the same gadoxetate DCE-MRI assay could allow quantitative translational safety models to predict toxicity in humans [52]. This information will be important for pharmaceutical companies and regulatory agencies when assessing the translatability of preclinical hepatic safety studies.

In conclusion, this work has demonstrated that gadoxetate DCE-MRI is sufficiently sensitive to detect effects on hepatobiliary transporter proteins at the clinical dose of rifampicin, prior to a rise in liver injury markers. Pharmacokinetic modeling provides additional information estimating both Oatp1 and Mrp2 transporter function. Finally, the changes in modeled transporter functional parameters were robust between different laboratories and MR scanner field strengths. These results are a first step in the validation of gadoxetate DCE-MRI as a biomarker of hepatobiliary transporter function that may be of value in the evaluation of drug safety, and may ultimately also be of use in clinical practice for patients. This study demonstrates that gadoxetate DCE-MRI is a sensitive and robust biomarker to detect early changes in hepatobiliary transporter function *in vivo* in rats prior to established biomarkers of liver toxicity.

## Supporting information

S1 Table *k*_*1*_, *k*_*2*_, maximum RE and AUC data from multicenter *in vivo* Study 3. (DOCX)

## Abbreviations

AUC: Area Under the Curve
DCE-MRI: Dynamic Contrast Enhanced Magnetic Resonance Imaging
DILI: Drug-induced liver injury
ICC: intraclass correlation coefficient
MR: Magnetic Resonance
Mrp2: multidrug resistance-associated protein 2
Oatp1: organic anion-transporting polypeptide 1
RE: Relative Enhancement
