# Supplementary material for: A multi-center preclinical study of gadoxetate DCE-MRI in rats as a biomarker of drug induced inhibition of liver transporter function
Source: PLoS One. 2018 May 17;13(5):e0197213. doi: 10.1371/journal.pone.0197213 (PMC5957399; doi:10.1371/journal.pone.0197213)
Supplement: S1 Table — (DOCX) [file pone.0197213.s001.docx]

**Supplementary Table 1.** ***k_1_*, *k_2_*, maximum RE and AUC data from multicenter *in vivo* Study 3**

|  |  | Vehicle | | Rifampicin | |
| --- | --- | --- | --- | --- | --- |
|  | Center | Mean | SEM | mean | SEM |
| *k_1_ (s^-1^)* | i | 34.9 | 6.4 | 7.1 | 1.2 |
|  | ii | 58.7 | 6.0 | 19.1 | 3.5 |
|  | iii | 29.2 | 2.0 | 10.7 | 1.3 |
|  | iv | 32.6 | 3.9 | 10.5 | 1.1 |
| *k_2_ (s^-1^)* | i | 1.45 | 0.14 | 0.91 | 0.12 |
|  | ii | 1.32 | 0.06 | 0.65 | 0.08 |
|  | iii | 1.39 | 0.08 | 1.01 | 0.05 |
|  | iv | 1.94 | 0.19 | 1.18 | 0.22 |
| Maximum RE  (%) | i | 106.5 | 5.8 | 46.9 | 1.9 |
|  | ii | 150.0 | 3.9 | 49.0 | 4.8 |
|  | iii | 110.3 | 4.2 | 63.3 | 2.6 |
|  | iv | 75.6 | 4.9 | 37.6 | 3.5 |
| AUC | i | 16.5 | 1.4 | 11.6 | 0.8 |
|  | ii | 24.0 | 1.5 | 13.2 | 1.2 |
|  | iii | 17.8 | 0.94 | 14.3 | 0.7 |
|  | iv | 9.6 | 1.9 | 8.3 | 0.6 |
